# Deletion Extents Are Not the Cause of Clinical Variability in 22q11.2 Deletion Syndrome: Does the Interaction between DGCR8 and miRNA-CNVs Play a Major Role?

**DOI:** 10.3389/fgene.2017.00047

**Published:** 2017-05-01

**Authors:** Veronica Bertini, Alessia Azzarà, Annalisa Legitimo, Roberta Milone, Roberta Battini, Rita Consolini, Angelo Valetto

**Affiliations:** ^1^Cytogenetics and Molecular Genetics Unit, Department of Laboratory Medicine, Azienda Ospedaliera Univeristaria PisanaPisa, Italy; ^2^Laboratory of Immunology, Department of Clinical and Experimental Medicine, University of PisaPisa, Italy; ^3^Department of Developmental Neuroscience, Istituto di Ricovero e Cura a Carattere Scientifico Stella MarisPisa, Italy

**Keywords:** 22q11 deletion syndrome, array CGH, copy number variations, miRNA, CNV-miRNA, DGCR8

## Abstract

In humans, the most common genomic disorder is the hemizygous deletion of the chromosome 22q11.2 region, that results in the “22q11.2 deletion syndrome” (22q11.2DS). A peculiarity of 22q11.2DS is its great phenotypic variability that makes this pathology a classic example of a syndrome with variable expressivity and incomplete penetrance. The reasons for this variability have not been elucidated yet, and the molecular substrates underlying the different clinical features of 22q11.2DS are still debated. A cohort of 21 patients has been analyzed by array CGH in order to detect some of the genetic differences that may influence this variability. Two aspects have been investigated: (1) the precise localization of the deletion breakpoints within the low copy repeats (LCRs), (2) the additional Copy Number Variations (CNVs) elsewhere in the genome, by analyzing their gene content. Both protein-coding genes and miRNAs were considered, in order to discover possible epistatic interactions between genes of the 22q11.2 region and the rest of the genome. Eighteen out of twenty-one patients had a deletion of ~3 Mb mediated by LCR22-A and D, whereas 3/21 had a smaller deletion. The breakpoints within the LCR22-A and D do not have a major role in the phenotypic variability since they are rather clustered and the small differences concern genes that are not directly related to clinical signs of 22q11.2DS. A detailed analysis of the gene content of 22q11.2 deleted region indicates that this syndrome could be a bioenergetic disorder or consequence of an altered post-transcriptional gene regulation, due to the presence of *DGCR8*, a major player of the microRNA (miRNA) biogenesis. Only four genes with mitochondrial function are harbored in the additional CNVs, whereas 11 miRNA, all related to biological pathways present in the 22q11.2DS, have been detected in 19/21 patients. CNVs and miRNAs are new entities that have changed the order of complexity at the level of gene expression and regulation, thus CNV-miRNAs (miRNA harbored in the CNVs) are potential functional variants that should be considered high priority candidate variants in genotype-phenotype association studies. Deletion of *DGCR8*, the main actor in miRNA biogenesis, amplifies this variability. To our knowledge, this is the first report that focus on the miRNA-CNVs in 22q11.2DS, with the aim of trying to better understand their role in the variable expressivity and incomplete penetrance.

## Introduction

In humans, the most common genomic disorder is the hemizygous deletion of the chromosome 22q11.2 region that results in the “22q11.2 deletion syndrome” (22q11.2DS) (MIM #188400/#192430), also referred to as Di George, Velocardiofacial or Shprintzen syndrome.

A peculiarity of 22q11.2DS is its great phenotypic variability that makes this pathology a classic example of a syndrome with variable expressivity and incomplete penetrance.

22q11.2DS phenotype is characterized by a constellation of clinical signs, whose characterization has expanded considerably within the last decade. The phenotype includes many associated findings such as congenital heart disease (CHD; 50–75%), hypocalcemia (17–60%), immunological disorders (35–40%), palatal (69–100%), craniofacial (90%), skeletal (17–19%), gastroenterological (35%) anomalies and a range of neurocognitive, and psychiatric disorders (85%; McDonald-McGinn and Sullivan, 2011). The severity of symptoms is also variable, ranging from quite severe to near-normal life conditions (Bassett et al., 2011). 22q11.2 deletion can be transmitted by a normal parent with no clinically obvious phenotype, to an affected child (Leana-Cox et al., 1996) and this provides additional evidence that genetic modifiers, beyond the deleted genes themselves, play a key role in determining the 22q11.2DS phenotypic severity.

This variability can be due to stochastic and environmental processes as well as to genetic effects, with all these factors potentially acting alone or in combination. Currently, no mechanistic explanation exists, and the reasons underlying this phenotypic variability are still debated.

Extension of the deleted region could be a genetic cause underlying the phenotypic variability. Deletions in 22q11.2 region are a consequence of non-allelic homologous recombination (NAHR) due to misalignment of low copy repeats (LCRs) during meiosis (Edelmann et al., 1999; Kumar et al., 2008). Eight LCRs (named LRC22-A to H) have been identified, but only the four centromeric ones (LCR22-A to D) are implicated in this syndrome. Deletions mediated by different specific LCRs will result in different sets of genes being deleted and this is a cause of phenotypic variability. In any case, this does not play a major role, since over 90% of patients share a deletion between LCR22-A and LCR22-D.

Deletions that appear to have roughly the same size, since mediated by the same couple of LCRs, may exhibit variability at the sites of the NAHR event. A fine mapping of the breakpoints within the LCRs has been performed only in a few studies (Mantripragada et al., 2004; Urban et al., 2006; Bittel et al., 2009). These small differences of the breakpoint localization within the same LCR may influence the expression of genes that could have a major role in the clinical phenotype of 22q11.2DS.

The presence of additional CNVs in the rest of the genome could be another factor responsible for this variability. Human genome is much more genetically variable than previously appreciated. To date, nearly 10–15% of it has been annotated as copy-number variations (CNVs), namely microdeletions and microduplications, ranging from 1 kb to several Mb (Lee et al., 2007). Their presence may contribute significantly toward human phenotypic variability, complex behavioral traits, and disease susceptibility.

Epistatic interactions between genes in the 22q11.2DS region and other genes harbored in CNVs elsewhere in the genome could explain the variable expressivity and reduced penetrance of this syndrome.

According to the recent literature data, CNVs are a substantial risk factor for a range of neurodevelopment and psychiatric disorders such as autism, ADHD, schizophrenia, epilepsy, and intellectual disability (Kirov et al., 2009; Cooper et al., 2011; Vaishnavi et al., 2013).

In 22q11.2DS cases, a correlation between CNVs and schizophrenia has been explored (Williams et al., 2013). However, little is known about to the role of CNVs in modulating the outcome of other phenotypic aspects of this syndrome. Two recent articles focused on the role of CNVs as genetic modifiers involved in the variable cardiac phenotype, studying a very large cohort of 22q11.2 patients. Except for a common CNV, a *SLC2A3* duplication, that was significantly associated with CHDs (cardiac heart diseases), no differences were found in the number or in the size of common CNVs between 22q11DS cases with or without CHDs.

When rare CNVs were carefully examined for their gene content and function, specific cardiac networks appeared to be overrepresented in 22q11DS CHD cases but not in 22q11DS controls with no cardiac phenotype (Mlynarski et al., 2015, 2016).

In this view, we focused our attention on these two genetic factors as potential cause of variability, and, in particular (1) on the localization of the deletion breakpoints within LCRs, (2) and on the additional CNVs elsewhere in the genome, by analyzing their gene content. Both protein-coding genes and microRNAs (miRNAs) were evaluated, in order to discover possible epistatic interactions between genes of the 22q11.2 region and the rest of the genome.

## Materials and methods

### Subjects

Our study group consisted of 21 subjects with known 22q11.2 deletion who had been referred to our Institution for molecular-cytogenetic investigations (9 males, 12 females), with age ranging from to 3 months to 37 years. This study was approved by the Pediatric Committee of the Tuscany Region, Meyer Hospital, Firenze, Italy. An informed consent was signed prior to the genetic analyses. Main clinical aspects are reported in Table 1.

**Table 1:** **Clinical Findings in the 21 patients studied**.

					**Problems**							
**Patients**	**Age (years)**	**Phospho-calcium metabolism**	**Cellular immune deficiency**	**Autoimmunity**	**Cardiovascular**	**Gastrointestinal**	**Ear-nose-throat**	**Cognitive**	**Behavior**	**Neurological**	**Motoricity**	**Speech**
1	7.5		+	Psoriasis			Velopharyngeal insufficiency hearing impairment	+				+
2	37			Undifferentiated connective tissue disease			Velopharyngeal insufficiency		Anxiety disorder			
3	1.5	Neonatal hypocalcaemia	+		Ventricular sept defects, anomalies of aortic arch	Reflux, umbilical hernia						+
4	12		+	Alopecia				+				+
5	9			Thyroiditis		Cow's milk protein intolerance	Hearing impairment	+	Adjustment disorder, anxiety disorder	Panayiotopoulos syndrome		+
6	15.9							+		Panayiotopoulos syndrome	Hypotonia	+
7	34				Ventricular sept defects			+	Mood disorder			+
8	4										+	+
9	0.3				Ventricular sept defects patent foramen ovale							
10	17	Hypoparathyroidism	+	Thyroiditis				+		Epilepsy		
11	7	Neonatal hypocalcaemia + hypoparathyroidism	+		Ventricular sept defects patent foramen ovale	Inguinal hernia				Epilepsy		
12	11.8		+	Anti-nuclear antibodies	Fallot tetralogy							+
13	15.5	Neonatal hypocalcaemia	+		Ventricular sept defects	Feeding difficulties	Cleft palate hearing loss	+				+
14	13		+	Thyroiditis		Epigastralgia		+	Mood Disorder			
15	6.5		+		Ventricular sept defects, pulmunary stenosis			+			+	+
16	10.5		+					+				+
17	17		+		Ventricular sept defects, patent foramen ovale	Inguinal hernia	Sensorineural hearing loss	+				+
18	3				Fallot tetralogy		Velopharyngeal insufficiency					+
19	11	Hypocalcaemia	+		Aberrant subclavian artery	Gastroesophageal reflux	Bronchial stenosis	+	Mixed anxiety disorder			+
20	2, 5		+				Congenital subglottic stenosis					+
21	7, 5			Thyroiditis			Inferior turbinate hypertrophy + flat tympanogram	+	Autism spectrum disorder		+	+

*The age and the main clinical features of the patients are reported. +, indicates the presence of a clinical aspect*.

### Array-CGH

Genomic DNA of the patients was isolated from peripheral blood by standard methods; DNA from healthy subjects (a male and a female) was used as controls (Agilent Technologies, Santa Clara, California, USA). Five-hundred nanograms of genomic DNA both from the patient (test sample) and the control (reference sample) were differentially labeled with Cy5-dCTP or with Cy3-dCTP using random primer labeling according to manufacturer's protocol (Agilent). The labeling reactions were applied to the oligo-arrays and incubated for 24 h at 67°C in an oven. Slides were washed and scanned using the Agilent scanner, and the identification of individual spots on scanned arrays and quality slide evaluation was performed using the Agilent dedicated software (Feature Extraction, Agilent).

The array CGH was performed on 180K SurePrint G3 Human CGH Microarray (Agilent), that have a 13 KB overall median probe spacing (11 KB in Refseq genes). In order to better define the deletion breakpoints, we have also used 60K SurePrint G3 Unrestricted CGH ISCA v2, that has a 60 KB overall median probe spacing but higher in pathological or gene rich regions.

Given that the quality of the experiment may greatly influence the CNVs analysis (number and type of calls, probes included/excluded from a call), in order to have more powerful and clean data, we elaborated only those experiments that met the “excellent” criteria as determined by the QC report (Cytogenomic software, Agilent). In particular, the Derivative Log Ratio Spread (DLRS) was the main value considered for further analysis of the data: when >0.16, the experiment was discarded and repeated. CNVs were identified with Cytogenomics 3.0.6.6. (Agilent), using the Aberration Detection Method-2 (ADM-2) algorithm. This algorithm identifies aberrant intervals in a sample that has consistently high or low log ratios based on their statistical score. The score represents the deviation of the weighted average of the normalized log ratios from its expected value of zero and incorporates quality information about each probe measurement. ADM-2 uses an iterative procedure to find all genomic intervals with a score above a user/specified statistical threshold value. The threshold was set to a minimum of six with the minimum number of three probes required in a region and a minimum absolute log ratio of 0.25. In particular, we have examined the 22q11.2 region, focusing on probes delimiting the breakpoints, together with all the CNVs meeting the statistical parameter described above. We analyzed all the CNVs >3 contiguous probes for deletions and >4 probes for duplications, independently of their absolute size; they were compared to those reported in the http://dgv.tcag.ca/dgv/app/home. The gene content was established by UCSC Genome Browser (http://genome.ucsc.edu/) (NCBI37/hg19 assembly) and the gene function by RefSeq (https://www.ncbi.nlm.nih.gov/refseq/rsg/).

### Retrieval of CNV-microRNAs and target prediction

The miRNA content in CNVs was identified by analyzing the chromosomal coordinates in UCSC Genome Browser.

The function of these miRNAs and their validated and putative targets were identified from the miRWalk database (http://www.umm.uni-heidelberg.de/apps/zmf/mirwalk/). miRWalk (Dweep et al., 2011) is a comprehensive database that provides information on predicted as well as validated binding sites of miRNAs on their target genes and the functional patterns they are part of. For the prediction of putative targets by miRWalk, filters were set and applied to identify the 3′UTR that has a minimum seed match of seven nucleotides and retained only the targets predicted by multiple databases (consistently predicted by miRanda, miRDB, miRWalk, and TargetScan). This integration between multiple databases improves the accuracy or coverage of predictions by balancing out the precision and recall.

The biological pathways of genes targeted by these miRNA were analyzed with Gene Ontology (GO; http://www.geneontology.org/). GO has developed three structured controlled vocabularies to describe gene products in terms of their associated biological processes, cellular components and molecular functions (Ashburner et al., 2000). All the related functions associated with their enrichment scores and *p*-values derived with the multiple test adjustment set were done by Benjamini and Hochberg. Only the results with a corrected *p* < 0.05 were considered to be significant. For each miRNA, significative data on the biological process (GOBP) were imported into a Microsoft Excel Table.

## Results

**Figure 1:**
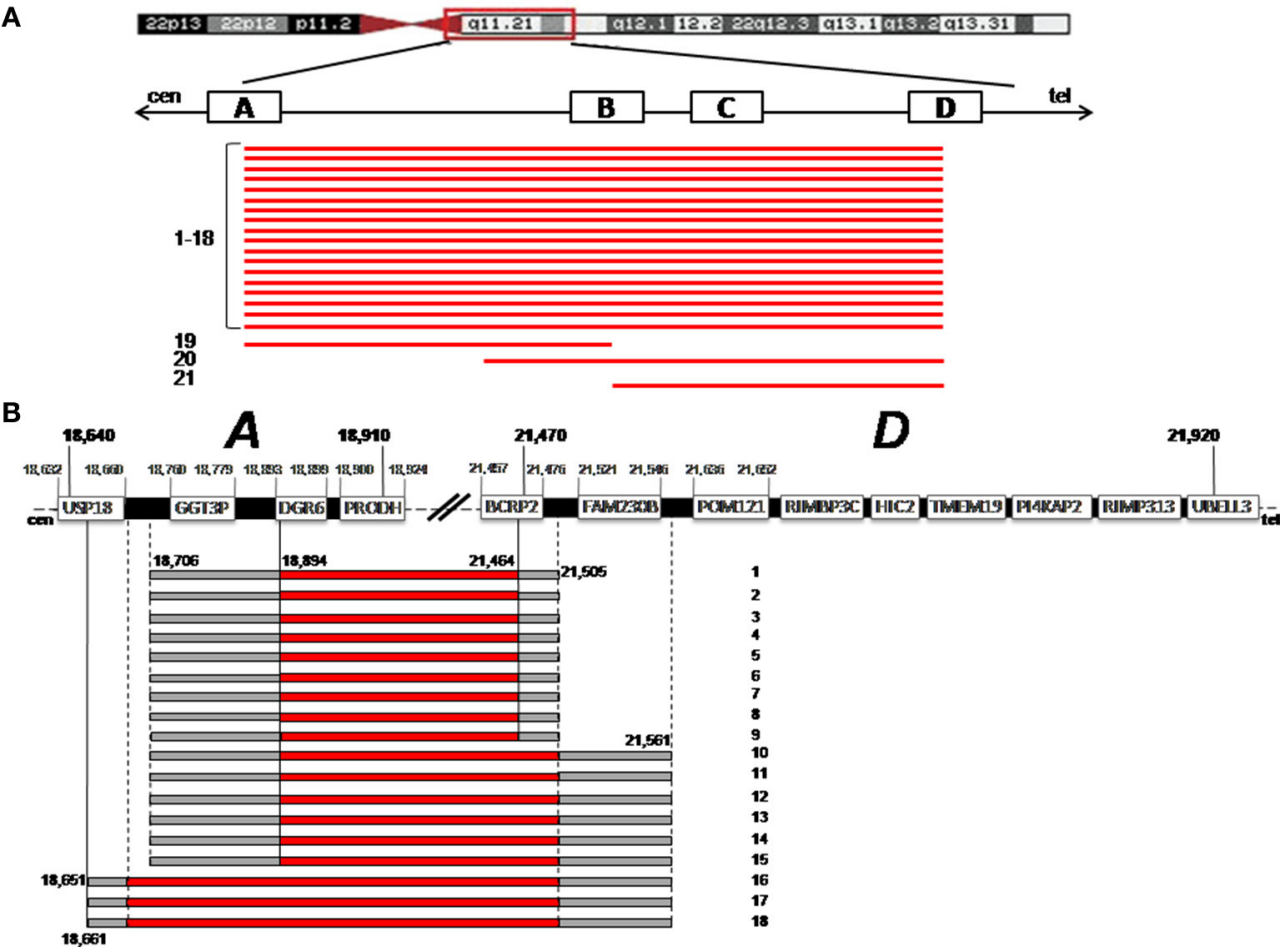
**(A)** The deletion extent is shown for the 21 patients (bottom, black bars). On the top, a schematic representation of chromosome 22 is given. The four Low Copy Repeats A to D (LCRA-D) involved in the deletion are indicated with white blocks. Cen is centromere; Tel is telomere. **(B)** Precise definition of the breakpoints is shown for patients 1–18. On the top, a detailed structure of LCR A–D is shown, with the genes harbored and their positions (hg19 Genome Build). The dark bars indicates the region deleted, the gray bars indicate the region that could or could not be deleted, with the probe positions.

We analyzed 21 subjects with known 22q11.2 deletion using the Agilent 180K whole genome and the 60K ISCA microarrays. A scheme of the deletion extent is reported in Figure 1A. Eighteen out of twenty-one patients had a 22q11.2 deletion of ~3 Mb mediated by LCR22-A and D, whereas 3/21 had smaller deletions, of 1.3 Mb (pt. 19), 2.4 Mb (pt. 20), and 745 kb (pt. 21), respectively.

In the 18 patients with the common ~3 Mb deletion, using the above sets of arrays, we were able to accurately define the deletion boundaries. LCR22-A and D extend for 270 and 450 Kb, respectively, and harbor many genes (Figure 1B); the “gray zone” (i.e., the region comprised between the last non-deleted oligo and the first deleted one) was restricted to a small interval. As far as the LCR22-A breakpoint is concerned, in three cases (pt n.16,17,18) *USP18* (Ubiquitin specific peptidase 18) was the most proximal deleted gene with breakpoint boundaries limited to a 10 Kb interval. In all the other patients, *DGCR6* is the first functional gene involved in the deletion since *GGT3P* (Gamma-glutamyltransferase 3 pseudogene) is a pseudogene.

As far as the LCR22-D breakpoint is concerned, *FAM230B* (Homo sapiens family with sequence similarity 230 member B) is not deleted in patients 1–9, whereas it is in the “gray zone” in the other nine cases (pts. 10–18). *FAM230B* is a long non-protein coding RNA, with unknown functions. *BCRP2* (breakpoint cluster region pseudogene 2) that may represent a difference in patients 1–9, is a pseudogene.

In the above 18 patients, the deleted region encompasses about 50 RefSeq genes, whose function has been well-established only in a small proportion. There is an abundance of non-coding RNA (a total of 13, 6 of which are miRNA) and nine genes (*MRPL40, PRODH, SLC25A1, TXNRD2, T10, ZDHHC, COMT, UFD1L, DGCR8*) that code for proteins, either localized to mitochondria or connected to mithocondrial functions. *DGCR8* (Di George Critical Region Gene 8), in particular, is a gene that also has a primary role in miRNA biogenesis.

### CNVs

In our cohort, besides the 22q11.2 deletion, array CGH showed the presence of additional CNVs. All the CNVs detected are reported in Table 2, independently of their size, position, or gene content; CNVs spanning segmental duplications were included, since they are often gene rich. Both “rare” (when not reported in normal population studies or with a frequency of <1% in a previously published control populations) and “common” (reported in several studies or with a frequence equal or higher than 1% in the control populations) were considered (Pinto et al., 2007; Vogler et al., 2010; Cooper and Mefford, 2011; Cooper et al., 2011; Coe et al., 2014).

**Table 2:** **Additional CNVs detected in the 21 patients are shown**.

**Chr**	**Band**	**Start**	**Stop**	**Size**	**1**	**2**	**3**	**4**	**5**	**6**	**7**	**8**	**9**	**10**	**11**	**12**	**13**	**14**	**15**	**16**	**17**	**18**	**19**	**20**	**21**	**Genes**
1	p36.22	9.332.851	9.389.984	57.133																						SPSB1
1	p36.21	12.846.834	12.912.625	65.791																						PRAMEF1, PRAMEF11, HNRNPCL1, HNRNPCL3
1	p36.13	16.840.487	17.252.023	411.536																						MIR3675, NBPF1, MST1L, LOC729574, CROCC
1	p33	49.921.976	49.988.135	66.159																						AGBL4
1	p32.2	58.291.803	58.409.467	117.664																			**x**			DAB1, DAB1-AS1
1	p21.1	104.098.248	104.307.708	209.460																						AMY1A, AMY1B, AMT1C, AMY2C, AMY2B
1	q21.2	149.041.933	149.243.967	202.034																						LOC101929780, NBPF23
1	q21.3	152.556.449	152.586.281	29.832																						LCE3C
1	q22	155.185.278	155.212.375	27.097																						GBA
1	q31.3	196.756.231	196.799.302	43.071																						CFHR3, CFHR1
1	q31.3	196.799.302	196.954.752	155.450																						CFHR3, CFHR4, CFHR2, CFHR5
1	q44	248.727.929	248.785.526	57.597																						OR2T34, OR2T10
2	p16.2	53.107.010	53.235.367	128.354																						–
2	p12	82.206.409	82.293.681	87.272																						–
2	p11.2	87.392.136	87.880.871	488.735																						LINC00152
2	p11.2	89.980.667	90.248.715	268.048																						–
2	q37.1	234.191.549	234.363.450	171.901					**x**																	ATG16L1, SCARNA6, SAG, DGKD
2	q37.3	242865920	243028452	162.532																						–
3	p21.31	46.804.379	46.848.728	44.349																						–
3	q29	195.419.168	195.472.855	53.687																						MIR570, MUC20
4	q12	57.059.830	57.375.375	315.545								**x**														KIAA1211, AASDH, PPAT, PAICS, SRP72, ARL9
4	q13.2	69.392.545	69.462.438	69.893																						UGT2B17
4	q13.2	70.156.093	70.259.782	103.689																						UGT2B28
4	q22.1	92.316.734	92.425.626	108.892														**x**								CCSER1
4	q22.1	93.554.942	93.575.642	20.700																						GRID2
4	q35.1–q35.2	187.021.294	187.542.770	521.476								**x**														FAM149A, FLJ38576, CYP4V2, KLKB1, F11, F11-AS1, MTNR1A, FAT1
5	p15.33	684.829	777.000	92.171																						TPPP
5	p15.33	723.194	820.424	97.230																						ZDHHC11
5	p15.2	12.686.815	12.734.149	47.334																						LINC01194
6	p25.3	328.710	378.956	50.246																						DUSP22
6	p22	29.093.912	29.140.640	46.728																						–
6	p22.1	29.854.870	29.896.710	41.840																						HCG4B
6	p21.33	31.310.027	31.326.021	15.994																						HLA-B, MIR6891
6	p21.32	32.480.027	32.521.929	41.902																						HLA-DRB5
6	p21.32	32.521.870	32.565.064	43.194																						HLA-DRB1
6	q14.1	78.979.172	79.023.328	44.156																						–
7	p14.1	38.296.176	38.352.444	56.268																						TARP
7	q11.22	70.274.285	71.725.368	1.451.083								**x**														WBSCR17, MIR3914-1, MIR3914-2, CALN1
7	q11.22–q11.23	72.003.013	72.315.358	312.345																						TYW1B, MIR4650-2
7	q11.23	76.139.282	76.511.836	372.554																						UPK3B, LOC100133091, POMZP3
7	q31.1	110.879.586	111.149.166	269.580																						**IMMP2L**
8	p23.1	6.939.251	7.753.583	814.332																						LINC00965, FAM66B, DEFB, USP17L1P, ZNF705G, SPAG2B, PRR23D1-2
8	p22	15.952.011	16.021.744	69.733																						MSR1
9	p11.2	43.686.924	43.836.428	149.504																						CNTNAP3B
9	q33.1	119.568.377	119.613.299	44.922														**x**								ASTN2
10	p12.1	27.613.431	27.695.910	82.479																						PTCHD3
10	q11.21	45.247.685	45.349.813	102.128																						TMEM72-AS1
10	q11.22	46.976.157	47.702.587	726.430																						GPRIN2, LOC100996758, NPY4R, LINC00842, FAM25G, AGAP9, ANXA8, ANTXRL
10	q21.3	68.154.487	68.251.594	97.107																						CTNNA3
10	q26.3	135.254.039	135.377.532	123.493																						SCART1, CYP2E1, SYCE1
11	p15.4	4.806.986	4.893.728	86.742																						OR52R1, OR52F2, OR52S1
11	p15.1	18.949.929	18.958.940	9.011																						MRGPRX1
11	q11	55.377.910	55.450.788	72.878																						OR4P4, OR4S2, OR4C6
11	q25	134.353.814	134.711.672	357.858																						LOC283177
12	p11.21	31.281.798	31.939.023	657.225																						FAM60A, FLJ13224, DENND5B, DENND5B-AS1, METTL20, AMN1
12	q24.12–q24.13	112.184.121	112.308.929	124.808																						**ACAD10**, ALDH2, MIR6761, MAPKAPK5, MAPKAPK5-AS1
12	q24.33	133.433.149	133.494.621	61.472																			**x**			CHFR
13	q21.1	55.674.811	55.875.862	201.051															**x**							MIR5007
14	q11.2	19.376.762	20.414.232	1.037.470																						OR11H12, POTEG, LOC642426, POTEM, OR4Q3, OR4M1, OR4N2, OR4K1-2–5
14	q24.3	74.001.651	74.022.324	20.673																						HEATR4, ACOT1
15	q11.1– q11.2	20.432.851	22.558.756	2.125.905																						GOLGA6L6, GOLGA8CP, POTEB2, CT60, LOC727924, OR4M2
15	q14	34.667.002	34.806.953	139.951																						GOLGA8A, MIR1233-1, MIR1233-2
15	q14	35.684.649	35.708.638	23.989																						DPH6
15	q15.3	43.895.645	43.988.818	93.173																						STRC, CATSPER2, **CKMT1A**
16	p13.11	15.048.751	15.110.727	61.976																						PDXDC1
16	p11.2– p11.1	32.573.808	33.625.989	1.052.181																						TP53TG3
16	p11.2–p11.1	33.625.989	35.147.508	1.521.519																						LOC283914, FLJ26245
16	q22.1	70.152.776	70.193.942	41.166																						PDPR
17	q12	34.437.475	34.817.481	380.006																						TBC1D, CCL3L3, CCL3L
17	q21.31	44.221.802	44.345.038	123.236																						KANSL1, KANSL1-AS1
17	q22	55.636.444	55.941.451	305.007																**x**						MSI2, CCDC182, **MRPS23**, CUEDC1
17	q25.3	77.372.621	77.392.578	19.957																						RBFOX3
18	q22.3–q23	73.098.243	73.200.515	102.272										**x**												SMIM21
21	q22.3	42.870.021	42.937.603	67.582																						TMPRSS2
22	q11.22	23.056.562	23.245.888	189.326																						MIR650, IGLL5
22	q11.23	24.347.959	24.390.254	42.295																						LOC391322, GSTT1
22	q11.23–q12.1	25.664.618	25.911.651	247.033																						IGLL3P, LRP5L, MIR6817
22	q13.1	39.359.112	39.385.485	26.373																						APOBEC3A, APOBEC3B
22	q13.2	42.792.565	42.945.064	152.499																						NFAM1, RRP7A
X	q22.2	103.186.126	103.303.380	117.254																						MIR1256, H2BFWT, H2BFM
X	q29	154396991	154425684	28.693																						–

*The chromosome (Chr), the cytogenetic band, the genomic position and the size are indicated. Blue boxes indicate a duplicated CNVs, red boxes deleted CNVs; yellow boxes are “rare” CNVs where the blue X indicates a duplication, and a red X a deletion. On the right size, the gene content is shown (OMIM genes; red, miRNA genes; blue, mitochondrial genes; bold black, non-coding RNA; brown, –indicates that no genes are present)*.

The average number of CNVs per patient was 11; these CNVs are present on all the chromosomes (except chromosome 19 and 20), with a size ranging from 9 kb to 2.1 Mb (average size 249 kb), and include roughly the same number of deletions and duplications.

Among the 81 CNVs, 11 were rare, detected in 7 patients (Table 2).

As far as their gene content is concerned, at first glance it emerges that among the total amount of 177 RefSeq, olfactory receptor, immunoglobulin family, HLA, and amylase genes are the most represented, as expected. Fourteen genes, both in common than in rare CNVs, are reported in the OMIM database (https://www.omim.org/); four genes related to mitochondrial function (*IMMP2L, ACAD10, MRPS23, CKMT1A)* were detected. Interestingly, among the other genes, there is a preponderance of miRNA (11). Nineteen out of 21 patients present at least one CNV-miRNA; some miRNAs are present only in deleted CNVs (i.e., miRNA6891, miRNA4650-2, miRNA5007, miRNA12331-1, miRNA1233-2), some only in duplicated CNVs (miRNA3914-1, miRNA3914-2, miRNA6761, miRNA650, miRNA6817, miRNA1256) and some in both (miRNA3675, miRNA570; Tables 2, 3).

**Table 3:** **The MiRNAs contained in the CNVs are shown in details**.

**Cytogenetic band**	**1**	**3**	**4**	**5**	**6**	**7**	**8**	**9**	**10**	**11**	**12**	**13**	**14**	**15**	**16**	**17**	** 18**	**19**	**21**	**miRNA**
1p36.13																				mirRNA3675
3q29																				miRNA570
6p21.33																				miRNA6891
7q11.22							**x**													miRNA3914-1, miRNA3914-2
7q11.22–q11.23																				miRNA4650-2
12q24.12–q24.13																				miRNA6761
13q21.1														**x**						miRNA5007
15q14																				miRNA1233-1, miRNA1233-2
22q11.22																				miRNA650
22q11.23–q12.1																				miRNA6817
Xq22.2																				miRNA1256

*The cytogenetic band, the patient number, and the miRNA are indicated. Red box, deletion; Blue box, duplication; Yellow box, rare CNVs where the blue X indicates a duplication, and a red X a deletion*.

### Functional pathways of miRNA

**Figure 2:**
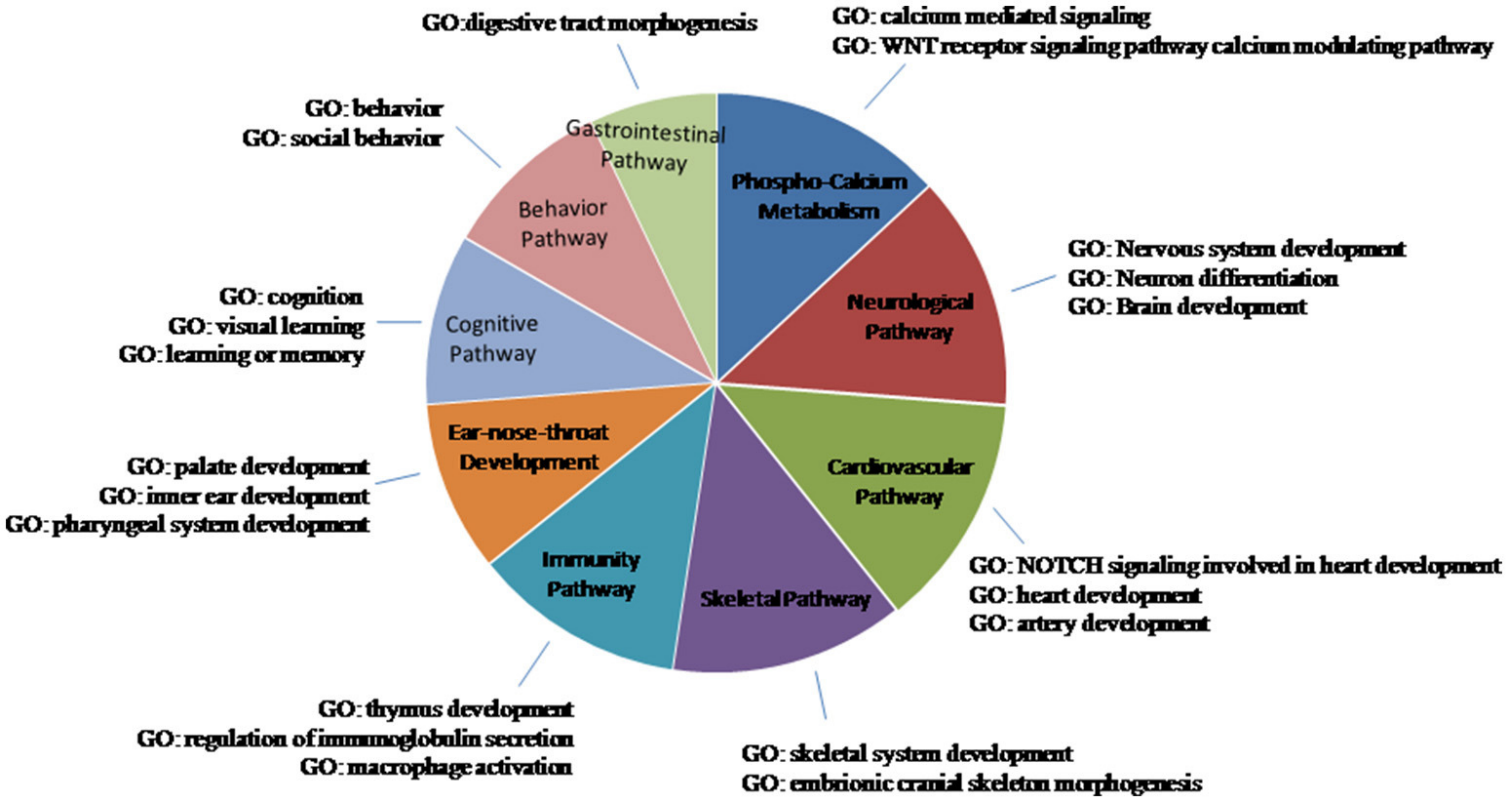
**Schematic representation of the biological pathways of the miRNA-CNVs**. GO is the Gene ontology process. Only the results with a *p* < 0.05 were considered to be significant. Many entries were related to clinical features of 22q11.2DS; they were grouped them into nine macro-areas.

These miRNAs regulate a large number of target genes, as indicated by the miRWalk database (Dweep et al., 2011; more than 400 entries, data not shown). Analyzing the biological pathways with GO, only those significant (*p* < 0.05) were considered. Many entries are related to clinical features of 22q11.2DS and we have grouped them into nine macro-areas, including: phospho-calcium metabolism; neurological, cardiovascular, immunity, cognitive-behavior, and gastrointestinal pathways; ear-nose-throat, skeletal development. As can be seen in Figure 2 and in Table 4, each miRNA has a regulatory role on the genes that are involved in the vast majority of these macro-areas.

**Table 4:** **Gene Ontology Biological Process (GOBP) of CNV-miRNA, with relation to the 22q11.2 deletion syndrome clinical signs**.

**miRNA**	**Phospho-calcium metabolism**	**Neurological pathway**	**Cardiovascular pathway**	**Skeletal pathway**	**Immunity pathway**	**Ear-nose-throat development**	**Cognitive pathway**	**Behavior pathway**	**Gastrointestinal pathway**
miRNA 3675	X	X	X	X		X	X	X	X
miRNA 570	X	X	X	X	X	X	X		X
miRNA 6891	X	X	X	X	X	X	X	X	
miRNA 6761	X	X	X	X	X		X	X	X
miRNA 650	X	X	X	X	X	X		X	
miRNA 1256	X	X	X	X	X				
miRNA 3914 1-2	X	X	X	X	X			X	
miRNA4650-2	X	X	X	X	X	X	X		X
miRNA 5007	X	X	X	X	X	X	X	X	
miRNA 6817	X	X	X	X	X	X	X	X	X
miRNA 1233	X	X	X	X	X	X	X	X	X
**TOT**	**11**	**11**	**11**	**11**	**10**	**8**	**8**	**8**	**6**

*The X indicates that a miRNA takes part to that process (p < 0.05)*.

## Discussion

As known, the clinical phenotype of 22q211.2DS is very variable and it can be considered the prototype of those syndromes with variable expressivity and reduced penetrance. The reasons for this variability have not been elucidated yet, and the molecular substrates underlying the different clinical features of 22q11.2DS are still debated.

In consideration of this, we collected a cohort of 21 patients and analyzed them by applying very homogeneous and strict criteria, in order to detect some of the genetic differences that may influence this variability. Two aspects were investigated: (1) the precise breakpoint localization and (2) the additional CNVs elsewhere in the genome in order to elucidate the possible interactions between their genes and those in the 22q11.2 region.

### Breakpoint localization

According to the literature, it is known that the vast majority of 22q11.2 deletions happen between LCR22-A and D, giving rise to the “common” ~3 Mb deletion. Also in our cohort, 18/21 pts have the deletion between A and D, whereas three cases have smaller deletions (Figure 1B).

In the 18 patients sharing the ~3 Mb deletion, the fine mapping of the breakpoints within LCR22-A and D allowed us to evaluate whether small differences of the gene content at the boundaries may play a role in the clinical variability of 22q.11.2 DS. Since LCR22-A and D are quite large and harbor many genes, it is expected that a certain variability could be present at the deletion sites. However, the combination of two array CGH platforms surprisingly showed that in our cohort the breakpoints localization is rather clustered. The only difference concerns the *USP18* and *FAM230B* genes, at the proximal and at the distal breakpoint, respectively.

*USP18* (NM_017414) codes for a protein that belongs to the ubiquitin-specific proteases (UBP) family of enzymes that cleave ubiquitin from ubiquitinated protein substrates. Mice lacking this gene are hypersensitive to interferon; in humans, no data are available about its loss-of-function. *FAM230B* (Homo sapiens family with sequence similarity 230 member B) is a long non-protein coding RNA, the function of which is unknown.

Even if our cohort is limited, it emerges from these data that the breakpoints within the LCR22-A and D are clustered and the small differences concern genes that are not directly related to clinical signs of 22q11.2DS, thus it can be inferred that the breakpoint localization does not have a major role in 22q11.2DS phenotypic variability.

### Additional CNVs and their gene content

22q11.2DS patients show a wide spectrum of clinical signs, but single aspects are recurrent (i.e., cardiological 50–75%, immunological 35–40%, neurobehavioral 85%), and cannot be related only to the presence of rare CNVs, which are defined as having a frequency equal or <1% in control populations. For this reason, in the present study, analysis of the gene content was not limited to the rare CNVs, but was extended also to the common ones. In Table 2, all the 81 CNVs detected in our cohort are reported, regardless of their size or frequency, and their genes content is shown.

Before discussing these results, it is worth noting that additional CNVs represent a common background in all the microdeletion syndromes, but most of them do not show the phenotypic variability seen in the 22q11.2DS. If we assume that CNVs could have a major impact on clinical variability, we should address the reason why CNVs affect the 22q11.2DS phenotype more severely than other microdeletion syndromes.

To find the genetic peculiarities that could justify such a preponderant role of CNVs only in 22q11.2DS, first of all we focused on the functional role of genes harbored in the 22q11.2 deleted region.

#### Peculiarity of the genes in the 22q11.2 region

The ~3 Mb region encompasses about 50 RefSeq genes; many of them have not been functionally well-characterized and little is known about their contribution to single clinical aspects of 22q11DS. However, at first glance, the region contains a large number of genes related to mitochondrial functions, and an abundance of non-coding RNA (a total of 13, 6 of which are miRNA) together with the presence of *DGCR8* (Di George Critical Region Gene 8), a gene with a primary role in miRNA biogenesis.

##### Mitochondria and embryonic development

During the embryo differentiation and early postnatal development, cells actively divide, and these divisions require a great amount of energy, with intense mitochondrial activity; it is expected that deficits in the bioenergetic machinery could set the basis for pleiotropic effects in the majority of developing organs and tissues.

Nine genes in 22q11.2 region code for proteins which are either localized in the mitochondria or are connected to mitochondrial functions (*MRPL40, PRODH, SLC25A1, TXNRD2, T10, ZDHHC, COMT, UFD1L, DGCR8*) (Meechan et al., 2011). 22q11.2DS is clearly due to an altered embryonal development that compromises early morphogenesis in the pharyngeal arches, heart, skeleton, and brain and thus it could be the consequence of metabolic and energetic problems, rather than of a decreased dosage of gene with a morphogenetic role.

Among the additional CNVs, four genes with mitochondrial function (*IMMP2L, ACAD10, MRPS23*, and *CKMT1A*) have been found. Even if our cohort is limited and it is not possible to demonstrate a correlation between clinical variability and mitochondrial pathways, a search for genes involved in mitochondria has not been performed yet, and it should deserve further attention in the future.

##### DGCR8 and CNV-miRNAs

*DGCR8* has a primary role in miRNA biogenesis (Han et al., 2004). MiRNAs exert a precise control over the spatiotemporal expression of individual genes as well as large gene networks. The way these tiny post-transcriptional gene regulators function is not yet fully known. There is a functional redundancy, since a single miRNA may have multiple mRNA targets, and a target may be also regulated by multiple miRNAs. *DGCR8* is a main actor in miRNA biogenesis, but alternative pathways are known (Berezikov et al., 2007). Variations in miRNA genes can severely affect downstream-regulated genes and their pathways.

As expected, haploinsufficency of *DGCR8* interferes with the processing of many miRNAs, and it is clearly emerging that some of phenotypic features of the 22q11.2DS could be ascribed not only to haploinsufficiency of coding genes but to an altered dosage of miRNAs.

In a knock-out mouse model, *Dgcr8* hemizygosity causes a 20–70% reduction in a subset of miRNAs in prefrontal cortex and hippocampus with a consequent dysregulation of the mRNA targets. *Dgcr8*^+/−^ mice show behavioral and cognitive dysfunction (Stark et al., 2008; Fénelon et al., 2011), present also in the human 22q11.2DS (Schofield et al., 2011; Ouchi et al., 2013). *Dgcr8* is essential for miRNA biogenesis in embryonic stem (ES) cells and regulates efficient ES-cell differentiation (Wang et al., 2007). Cardiomyocyte-specific deletion of *Dgcr8*^+/−^ demonstrated downregulation of a subset of mature cardiac enriched miRNAs; these mice experienced premature lethality due to cardiac failure, pointing out a critical role for miRNAs in maintaining cardiac function in mature cardiomyocytes (Rao et al., 2009).

More recently, the human miRNA expression pattern in the peripheral blood of 22q11.2DS has revealed a peculiar profile that differs from that of the controls; the deregulated miRNAs show a connection with the immunological, cardiac and hypocalcemic pathways (de la Morena et al., 2013; Sellier et al., 2014), suggesting that they may contribute to these phenotypic aspects in 22q11.2DS. Interestingly, processing of miRNAs is not globally altered in 22q11DS, but limited to the DGCR8 targets, providing compelling evidence of specific miRNA DGCR8-mediated dysregulation (Sellier et al., 2014).

In view of this, the role of the CNVs on the phenotypic variability of 22q11.2DS could be correlated non just to protein coding genes, but to miRNA gene content. Most of the studies that have dealt with CNVs and 22q11.2DS variability have been focused only on the protein coding genes, and the impact of altered dosage of non-coding regulatory miRNAs is largely unexplored.

Recently, some articles have highlighted a consistent co-localization of miRNA loci with CNV regions (CNV-miRNAs) (Marcinkowska et al., 2011; Veerappa et al., 2014). In a study on 1,715 subjects, about 5% of all miRNAs are harbored in the CNVs, ~9% of CNVs were shown to harbor miRNA genes and these CNV-miRNAs were identified in 83.8% individuals (Veerappa et al., 2014).

In our cohort, 11 CNV-miRNAs were identified (Table 3): as can be seen, some miRNAs are deleted, some duplicated, some others are present both in deletions and in duplications.

It seems obvious that the expression of miRNAs correlates with the CNV dose, as the expression of protein-coding genes depends on the copy number. CNV-miRNA with one allele duplicated (copy number = 3) or deleted (copy number = 1) is expected to have a level of expression roughly 1.5 or 0.5 times, respectively, compared to the wild type (copy number = 2). A change in a miRNA dosage will affect the expression of all of its target genes and may have a pleiotropic effect.

In 22q11.2DS patients, where DGCR8 activity is reduced by about 50% (Sellier et al., 2014), the expression pattern of CNV-miRNAs is completely dysregulated. The dosage of a miRNA in a deleted CNV is further decreased, whereas the dosage of miRNA in a duplicated CNV could be compensated by *DGCR8* hemizygosity. This dysregulation take place simultaneously on all miRNAs of an individual according to his CNV background.

It is worth noting that all the 11 miRNAs detected in the present cohort of patients have a biological role in pathways related to some phenotypic characteristics of 22q11.2DS (Table 4).

These changes in miRNAs expression are likely to contribute significantly toward 22q11.2DS phenotypic variability. Therefore, *DGCR8* could represent the genetic particularity according to which the additional CNVs affect the 22q11.2DS phenotype more greatly than other microdeletion syndromes.

In view of this, 22q11.2DS could be mainly related to a dysregulation of post-transcriptional modifications that lead to an altered dosage of proteins crucial for phenotype development.

## Concluding remarks

In this paper some of the genetic factors that can influence 22q11.2DS variability have been analyzed, and, even if the cohort of patients is limited, interesting observations can be made.

22q11.2 breakpoints are rather clustered, and it is unlikely that they play a major role in the variability.

A detailed analysis of the gene content of 22q11.2 deleted region indicates that this syndrome could be a metabolic disorder or consequence of altered post-transcriptional gene modifications.

In the additional CNVs, four genes with mitochondrial functions and 11 miRNAs, all related to the biological pathways present in the 22q11.2DS, have been detected in 4/21 and in19/21 patients, respectively.

CNVs and miRNAs are new entities that have changed the order of complexity at the level of gene expression and regulation, thus CNV-miRNAs are potential functional variants that should be considered high priority candidate variants in genotype-phenotype association studies. Deletion of *DGCR8*, the main actor in miRNA biogenesis, amplifies this variability.

To our knowledge, this is the first report that focuses on the CNV-microRNAs in 22q11.2DS, with the aim of trying to better understand their role in the variable expressivity and reduced penetrance.

In the near future, it will be important to characterize the pathways of these CNV-miRNAs in a much more comprehensive manner in order to improve the genetic networks that can contribute to the pathophysiology of 22q11.2.

## Author contributions

AV, VB, and RC participated in the design of this study. VB, AA, and AV performed the array CGH and the CNV gene content analyses; and contributed to writing the manuscript. AL and RC performed the immunological and the clinical characterization of the patients. RM and RB performed the clinical and neuropsychiatric investigations. All authors have read and approve the final version of this manuscript.

## Funding

This work has been supported by a fund (PRA_2015_0104) Progetto di Ricerca di Ateneo 2015 (PRA), Università di Pisa.

### Conflict of interest statement

The authors declare that the research was conducted in the absence of any commercial or financial relationships that could be construed as a potential conflict of interest.
